# Free vibration and buckling analysis of axially functionally graded tapered Timoshenko beams using B-spline-based isogeometric analysis

**DOI:** 10.1016/j.heliyon.2024.e41302

**Published:** 2024-12-17

**Authors:** Farzad Abdi, Aazam Ghasemi, Alireza Ariaei, S. Ali Eftekhari, Mehrdad Nasr, Mohamad Khaje Khabaz, Soheil Salahshour

**Affiliations:** a Department of Mechanical Engineering, University of Delaware, Newark, DE, 19716, USA; b Department of Mechanical Engineering, Najafabad Branch, Islamic Azad University, Najafabad, Iran; c Department of Mechanical Engineering, Faculty of Engineering, University of Isfahan, Isfahan, Iran; d Department of Mechanical Engineering, Khomeinishahr Branch, Islamic Azad University, Khomeinishahr, Iran; e Department of Solid Mechanic, Faculty of Mechanical Engineering, University of Kashan, Kashan, Iran; f Faculty of Engineering and Natural Sciences, Istanbul Okan University, Istanbul, Turkey; g Faculty of Engineering and Natural Sciences, Bahcesehir University, Istanbul, Turkey; h Faculty of Science and Letters, Piri Reis University, Tuzla, Istanbul, Turkey

**Keywords:** Buckling, Free vibration, Axially functionally graded material, Isogeometric, Tapered timoshenko beam

## Abstract

This study considers Timoshenko beam theory and the isogeometric analysis method to investigate the free vibration and buckling of axially functionally graded (AFG) tapered beams. The governing equations are obtained from the kinematic assumptions of Timoshenko beam theory and Hamilton's principle. The isogeometric analysis approach is implemented to solve the motion equations. One-dimensional B-spline basis functions are used to estimate the displacement field, describe the geometry, and illustrate the deformed shapes of the beam. Due to suffering the isogeometric approach from the shear locking phenomenon, the selectively reduced integration is applied. It is shown that this method can mitigate the effect of shear locking. In this attempt, the effect of material non-homogeneity parameters, mass density, Young's modulus, and taper ratio on the critical buckling loads and natural frequencies are considered for various boundary conditions. Several numerical examples show the accuracy and reliability of this method. The obtained results are in accord with the ones in the related articles and can be adopted as future reference solutions.

## 1 Introduction

Beams are the most common components applied in mechanical and civil structures like heavy motor vehicles, aircraft, bridges, and high-rise buildings [1,2]. AFG tapered beams facilitate achieving some optimization targets, such as the desired strength with the least material usage. For example, wind turbines are more effective in resisting loads by using fewer amounts of materials [3,4]. Due to this reason, usages of AFG tapered beams have become preferable in many engineering fields [5,6].

Two of the most-used beam theories are the Timoshenko and Euler-Bernoulli theories [7,8]. The displacement of the beam is considered without shear effects in Euler-Bernoulli's theory. Euler-Bernoulli overrates the natural frequencies of a beam, specifically for higher mode. Timoshenko developed his theory and formulated the shear effect, although the rotational inertia of thick beams was investigated by Rayleigh for the first time. This theory includes rotatory inertia and transverse shear deformation and presents a better result, especially for thick beams [9,10].

### 1.1 Buckling and free vibration of AFG Timoshenko beams

Functionally graded materials (FGMs) were designed as thermal barrier materials in Japan [11]. Since then, these materials have been of major importance among engineers and researchers due to their particular advantages like thermal resistance and high stiffness [12,13]. FGMs are made by varying the volume fraction of constituent material in any spatial direction. AFG materials have high engineering applications because of their ability to improve the distribution of weight and strength while the structural integrity is maintained [14,15]. Analysis of AFG tapered Timoshenko beams is more complicated in comparison with the homogeneous and uniform ones because of varying the cross-sectional parameters (i.e., the moment of inertia and area, along the beam axis). The material properties like Young's modulus, shear modulus, and mass density can change along the beam axis, leading to more complex governing equations [16]. Many researchers have suggested various methods to investigate AFG Timoshenko beams [[17], [18], [19], [20]]. Ghayesh and Farokhi studied the nonlinear bending and vibrations of tapered beams made of (AFG) material based on numerical methods [21]. They employed discretized via the Galerkin modal decomposition approach, to a large number of symmetric and asymmetric modes. In another attempt by Ghayesh, the coupled axial-transverse-rotational nonlinear forced vibrations of Timoshenko tapered beams made of an axially functionally graded (AFG) material subjected to an external harmonic excitation have been presented [22].

In this research, the Timoshenko beam theory (TBT) is used as the fundamental framework of the theoretical model. The study revealed that altering the shape of the object's cross-section from top to bottom had a more significant effect than changing its shape.

Shahba et al. [23] applied superconvergent element (SCE) shape functions in the finite element method (FEM) to assess the stability and free vibration of AFG tapered Timoshenko beams. In their work, the critical loads and natural frequencies are computed for different non-homogeneity parameters, taper ratio, attached mass, elastic supports, and various boundary conditions. Rajasekaran [24] analyzed the buckling and free vibration of the AFG tapered beams applying a differential transformation (DT) based on the dynamic stiffness approach. He considered both Timoshenko and Euler-Bernoulli's beam theories. He assessed free vibration and buckling of a rotating and non-rotating beam, which are influenced by taper ratio and rotary inertia.

Huang et al. [25] applied an auxiliary function to drive a single governing equation from the coupled governing equations of a Timoshenko beam. By applying the mode shapes as power series functions, the driven characteristic equation becomes a polynomial equation where the natural frequencies can be computed from the multi-roots. They presented the results for uniform, tapered, homogeneous, and AFG Timoshenko beams [26]. Closed-form polynomial functions for a specific class of AFG beams with a uniform cross-sectional area and clamped-clamped boundary condition were presented as a solution by Sarkar and Ganguli [27]. Gan et al. [28] investigated the dynamic response of non-uniform AFG Timoshenko beams under several moving point loads where the implicit Newmark method is adopted. They assessed the influence of the aspect ratio, the spacing between the multiple moving point loads, and the non-uniform cross-section on the dynamic responses of the beams. The free vibration of AFG beams whose material properties and geometry have stepped changes is studied by Bombill et al. [29]. They applied the differential quadrature method (DQM) together with the domain decomposition technique. Elastic boundary conditions are considered, and the results are demonstrated for various combinations of step location, material properties, and boundary conditions. Fang and Zhou [30] assessed the free vibration of rotating AFG tapered Timoshenko beams through the Chebyshev-Ritz method. They studied the influence of the material gradient, rotary inertia, speed ratio, taper ratio, and hub radius ratio on the natural frequencies for different boundary conditions. Singh and Sharma [31] applied a harmonic differential quadrature (HDQ) method to solve the motion equation. They assessed the influence of taper ratio, aspect ratio, and non-homogeneity parameters on the natural frequencies of the AFG tapered clamped–clamped beam. Akbaş [32] analyzed forced and free vibration responses of the AFG beam under a harmonic load by the Ritz method. His results showed that the effects of dynamic parameters, geometric parameters, and material graduation are significant on the vibration response.

The initial shapes and the precision in FEM are lost due to the errors originating from the geometric description and the discretization of the model [33]. However, Isogeometric analysis (IGA) can overcome this issue by integrating the FE analysis into the geometry model defined by the computer-aided design (CAD) without any shape approximation [34]. Using spline basis functions in CAD software causes arbitrary geometries as well as free-form shapes to be accurate [35,36].

### 1.2 Timoshenko beam modeling through the isogeometric method

As a logical extension to the FEM, Isogeometric analysis was introduced by Hughes et al. [37]. The purpose of this approach is to apply the spline functions used to define the geometry in the computer-aided design (CAD) as an interpolation function of the unknown physical variables instead of using Lagrange polynomials as the standard FEM [38]. Spline functions, like B-spline and Non-uniform rational B-spline (NURBS), guarantee C^p−1^ continuity, p being the degree of the spline, as opposed to the usual C^0^ continuity obtained with the standard finite element shape functions. It is determined that higher-order continuity between separate patches can extremely enhance computational results compared to standard finite element. Furthermore, studies on Isogeometric analysis have revealed that the high regularity properties of the spline functions improve the accuracy-to-computational-effort ratio and smaller absolute errors compared with standard FEM [[39], [40], [41]]. Alesad et al. investigated the 1D refined beam theories with the Isogeometric approach (IGA) for the static and free vibration analysis of beam structures. They used the B-spline basis functions utilized in IGA to approximate the displacement field due to their interesting attributes in the analysis [42].

A B-spline curved beam was used for analyzing negative stiffness structures by Ai et al. [43]. In this work, a static analysis model of a B-spline curved beam is utilized to evaluate the force-displacement relations of curved beams in different configurations. In addition, the configuration for the B-spline curved beam and the relation between the geometric parameters and the mechanical properties of the B-spline curved are presented [44,45].

Many authors have assessed the static and dynamic response of Timoshenko beams through the isogeometric approach. For instance, Lee and Park [46] investigated the free vibration of Timoshenko beams through the isogeometric method. Three refinement schemes are applied, and it is found that the k-refinement presents better results compared to the h- and p-refinements. They revealed that the boundary conditions could change the accuracy of the isogeometric solution and increase the number of elements and the degree of basis function, leading to the removal of the shear-locking phenomenon in a slender beam. The same authors [47] investigated the static behavior of Timoshenko beams for different boundary conditions. They compared full Gauss integration (FI) and selectively reduced integration (SRI) methods and found that SRI can efficiently mitigate the shear-locking phenomenon and improve the results. They illustrated their results for different refinement methods, the order of basis functions, the number of elements, and thickness-to-length ratios. Belgaid and Bouazzouni [48] introduced the isogeometric collocation method (IGC) and studied the free vibrations of concrete-steel composite Timoshenko beams. They compared their results with those obtained by the analytical and experimental methods. Cazzani et al. [36,49] applied the NURBS technique to model the geometry and displacement field of the plane historical masonry heritage arches and Timoshenko curved beams. They compared their results with other available ones in the related literature. They revealed that by selecting the proper basis functions' degree or the number of elements, one could overcome the membrane and shear locking phenomenon. Kiendl et al. [50] applied bending displacement as the only unknown field for the numerical formulation of Timoshenko beams. They expanded the strong and weak forms of the equations and developed Galerkin and isogeometric collocation formulations, which were completely locking-free. Gillebaart and De Breuker [51] coupled the isogeometric curved Timoshenko beam model and isogeometric potential flow model and combined these with a boundary-layer model. This method is implemented to optimize an active morphing airfoil. For symmetric and asymmetric bidirectional functionally graded materials (BDFGMs), Huynh et al. [52] investigated the free vibration characteristic of Timoshenko beams through the isogeometric method. They considered the distributions of material properties as a NURBS surface and defined the volume fraction of constituents by power and exponential laws. They compared their results with other papers and confirmed the accuracy of the IGA method. Huynh et al. [53] studied free vibration, buckling, and bending of transverse FGM curved beams through the isogeometric approach. They assessed circular, parabolic, elliptic, and cycloid beams of different boundary conditions and curvatures. The impact of aspect ratio, thickness-to-length ratio, and material distribution on the results is studied. Chen et al. [54] assessed the free vibration of transverse FGM beams with general boundary conditions. They depicted their results for different stiffness of spring, non-homogeneity parameters, and thickness-to-length ratios and assessed their effects on the natural frequencies. Hu et al. [55] studied transient response, natural frequency, and static bending of laminated Timoshenko curved microbeams by integrating the IGA method into the modified couple stress theory. They considered material coupling for laminated composites with the use of equivalent elastic modulus. The results considered the effects of boundary conditions, aspect ratios, and lay-ups. A surface elasticity theory and a modified couple stress theory (MCST) were implemented by Yin et al. [56] to develop a novel model in the IGA Timoshenko beam. Basis functions of NURBS efficiently fulfill the higher continuity that is needed in MCST. It is revealed that in very thin beams the effect of surface energy and microstructure should be considered.

Many authors have assessed the Timoshenko beam shear locking phenomenon in the isogeometric approach. To name a few, Echter and Bischoff [41] compared the convergence rate of the FEM and isogeometric method and realized that higher-order continuity between internal elements improves the results in the isogeometric method. They revealed that the isogeometric approach suffers from locking phenomena, and the discrete shear gap (DSG) method can conquer shear locking. Veiga et al. [57] and Auricchio et al. [58] applied the isogeometric collocation method by considering displacement-base and mixed formulations for Timoshenko beams and spatial Timoshenko rods, respectively. They proved that by applying mixed methods, locking-free solutions are achieved independent of the degree of the unknown fields. Bouclier et al. [59] adopted $\bar{B}$ projection and selective reduce integration methods to decrease shear and membrane locking. They assessed the static behavior of straight and curved Timoshenko beams and compared their results with those of the others. Furthermore, Liu et al. [60] adopted the mentioned methods to analyze out-of-plan and in-plan vibration of Timoshenko curved beams. To avoid the membrane and shear locking phenomenon, Adam et al. [35] applied the improved selective reduced integration for B-spline and NURBS finite element and evaluated their results for curved cantilever beams. For curved Timoshenko beams in Curvilinear coordinates, Choi and Cho [61] proposed an invariant formulation to overcome self-straining. They combined the proposed invariant formulation with $\bar{B}$ projection and selectively reduced integration methods to diminish membrane and shear locking. Hu et al. [62] applied multiple sets of lower-order basis functions. When adopting the order reduction (OR) method, the membrane and shear strains are characterized by the reduced order of basis functions.

Zheng et al. [63] presented a nonlinear finite element formulation of axially functionally graded(AFG) tapered microbeams, based on the modified couple stress and Euler–Bernoulli beam theory.They analyzed size-dependent nonlinear free vibration of AFG non-uniform microbeams by using finite element simulation.

However, there are still only rare studies on the free vibration and buckling of tapered Timoshenko beams, which have extensive usage in engineering applications. Within this context, the current work's major contribution is to utilize the B-spline-based isogeometric framework to model free vibration, and buckling of AFG tapered Timoshenko beam. Implementing the best computational features of the CAD and FEM, isogeometric analysis can be considered a powerful method to solve the derived equations. In this study, B-spline basis functions are applied rather than traditional Lagrange interpolation functions to estimate the displacement fields and characterize the geometry [3]. The major benefits of the B-spline-based isogeometric analysis over the conventional FEM are the capability of constructing exact geometries and using the piecewise C^p−1^ continuous basis functions, which present superior robustness and accuracy in comparison to FEM [64]. Furthermore, the present isogeometric beam element can determine precise mode shapes and natural frequencies of beams owing to the lack of any limitation on the choice of basis function's order [4].

The isogeometric approach can suffer from the shear locking phenomenon the same as FEM; however, it is demonstrated that selectively reduced integration can efficiently reduce the shear locking effect [65]. It is supposed that material properties vary with power-law formulations through the length of the beam, and three different tapering cases of the cross-sectional profile are considered. Some numerical cases are assessed to validate the precision of the IGA method and give an insight into the influence of the non-homogeneity parameter, Young's modulus, taper ratio, and mass density on the natural frequencies and buckling loads. The results obtained here are presented for different mentioned parameters and boundary conditions and are compared with other literature.

This paper is structured as follows: Section 2 is related to theoretical formulation in which a summary of B-spline basis functions and curves, free vibration and buckling isogeometric formulation, the geometry of the beam, and axial variation of material properties are presented. In section 3, the phenomena of shear locking and convergence of the proposed method are investigated. The numerical results of studying free vibration and buckling of the Timoshenko beam are demonstrated through several examples to elaborate the accuracy and performance of the current approach. Some conclusions drawn from the study are given in Section 5.

## 2 Theoretical formulation

### 2.1 B-spline basis functions and curves

A Knot vector is a sequence of non-decreasing real values in the parametric space defined as [66]:

(1) $$\Xi =\left\{\xi_{0},\xi_{1},\xi_{2},\ldots ,\xi_{p+n+1}\right\}$$

Where $\xi_{i}$ is the *i*th knot, *n+*1 is the number of basis functions and control points, and *p* is the order of B-splines [67]. A knot vector is called uniform if knots are spaced uniformly; otherwise, it is named non-uniform. Furthermore, it is named open (clamped) if the first and the last knots are repeated for *p*+1 times. Open, uniform knot vectors are applied throughout this study [68].

B-splines offer greater flexibility and smoothness in curve fitting and approximation compared to other methods like multiple scales due to their local control, which allows for precise adjustments without affecting the entire curve.

The B-spline basis functions, which are created from a knot vector, are applied in this study. The B-spline method offers advantages like smooth basis functions, which ensure higher continuity in the solution. This is particularly useful for vibration analysis, where the accuracy of higher modes is important. Compared to methods like the method of multiple scales, which are more suited for perturbation analysis of nonlinear problems, B-splines are more versatile for a wide range of linear and nonlinear problems, offering greater flexibility in refinement and adaptability. The *i*th B-spline basis function of degree *p* is represented by $N_{i,p}\left(\xi \right)$ and can be stated recursively as [69]:

(2a) $$N_{i,0}\left(\xi \right)=\left\{ \begin{array}{cc} 1 & \;\text{if}\;\xi_{i}\leq \xi \;<\xi_{i+1}\\ 0 & \;\text{otherwise} \end{array}\right.$$

(2b) $$N_{i,p}\left(\xi \right)=\frac{\xi -\xi_{i}}{\xi_{i+p}-\xi_{i}}N_{i,p-1}\left(\xi \right)+\frac{\xi_{i+p+1}-\xi }{\xi_{i+p+1}-\xi_{i+1}}N_{i+1,p-1}\left(\xi \right)$$

The B-spline functions are $C^{p-1}$ continuous at not repeated internal knots and are $C^{p-k}$ continuous if the knot has multiplicity *k*. A *p*th degree B-spline curve is formed by combining *p*th degree B-spline basis functions and control points in the following way:

(3) $$C\left(\xi \right)=\sum_{i=0}^{n}N_{i,p}\left(\xi \right)P_{i}$$

**Fig. 1:**
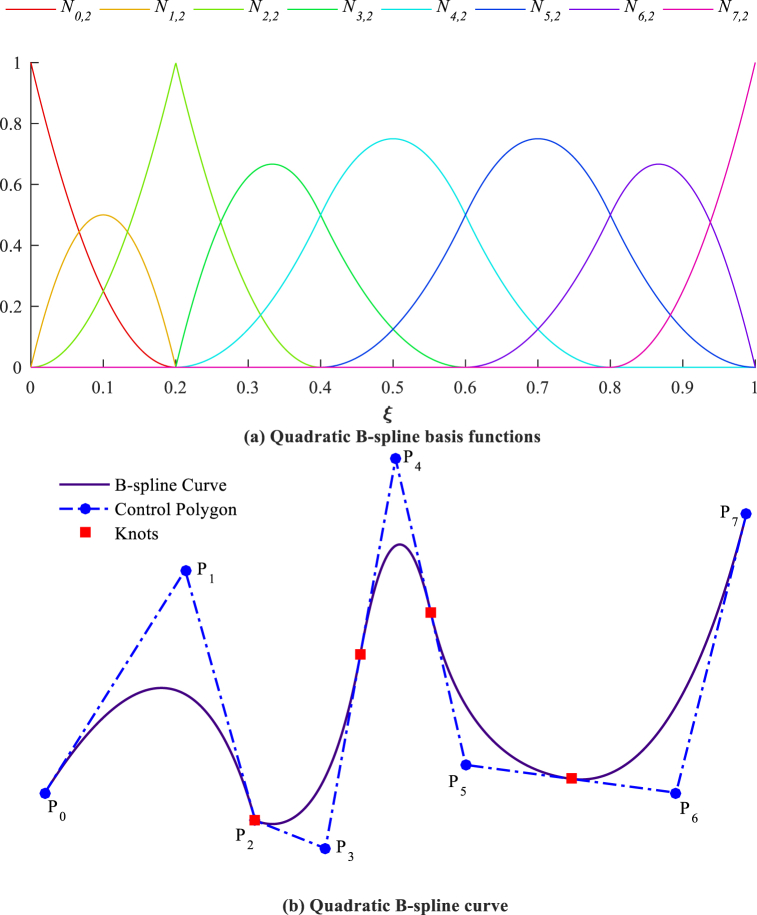
Quadratic basis functions and B-spline curve with knot vector Ξ={0,0,0,0.2,0.2,0.4,0.6,0.8,1,1,1}.

Where the control points are determined in physical space and construct a control polygon, and the knot vector is used to define the B-splines. Quadratic B-spline basis functions and a curve are presented in Fig. 1. More details about the CAD basis functions and their properties can be found in this reference [70].

### 2.2 The isogeometric formulation for free vibration and buckling

Based on the Timoshenko beam theory, the axial and transverse displacements can be stated as follows [45]:

(4a) $$\bar{U}\left(x,z,t\right)=-z\theta \left(x,t\right)$$

(4b) $$\bar{W}\left(x,z,t\right)=w\left(x,t\right)$$

**Fig. 2:**
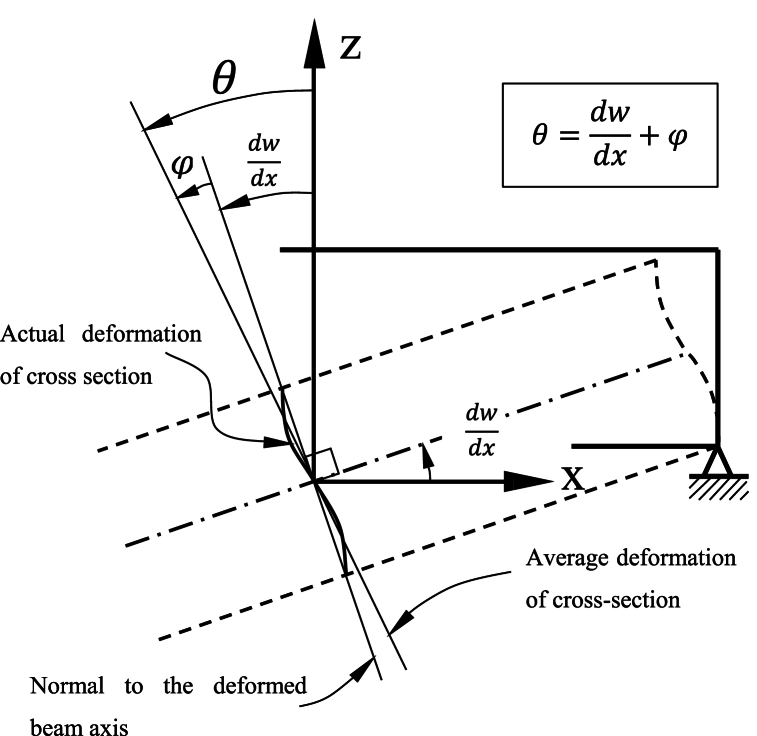
Rotation of cross-section in the Timoshenko beam theory.

Where *x* and *z* are the spatial coordinates, θ is the cross-section rotation, *w* is the transverse displacement, and *t* is time (Fig. 2). By applying Eq. (4), normal and transverse shear strains are defined as [71]:

(5a) $$\varepsilon_{xz}=-z\frac{\partial \theta }{\partial x}$$

(5b) $$\gamma_{xz}=\frac{\partial w}{\partial x}-\theta =-\varphi$$

The deformation of elements of the Timoshenko beam undergoes a rotation φ of the beam cross-section due to bending, and this rotation independent of the shear effect for all statically determinate beams [72]. The axial motion is often ignored in favor of transverse motion when it is relatively small or less significant compared to the transverse effects, though this assumption may need revisiting if material properties vary significantly along the axial direction, as it could affect the accuracy of the analysis. Concerning Hooke's law, normal and transverse shear stresses can be written as [73]:

(6a) $$\sigma_{xx}=E\left(x\right)\varepsilon_{xx}$$

(6b) $$\tau_{xz}=\kappa G\left(x\right)\gamma_{xz}$$

where κ is the shear correction factor, which is dependent on the cross-section profile. *E* and *G* are the Young's and shear modulus, respectively. Due to the axial variation of material properties in AFG beams, shear modulus and Young's modulus are the functions of the longitudinal coordinate. Based on the mentioned equations, the strain energy, kinetic energy, and work done by the axial load *P*_*0*_ can be written as:

(7a) $$U=\frac{1}{2}\int_{0}^{L}\int_{A}\left(\sigma_{xx}\varepsilon_{xx}+\tau_{xz}\gamma_{xz}\right)dAdx=\underset{U_{b}}{\underbrace{\frac{1}{2}\int_{0}^{L}\int_{A}\left(\sigma_{xx}\varepsilon_{xx}\right)dAdx}}+\underset{U_{s}}{\underbrace{\frac{1}{2}\int_{0}^{L}\int_{A}\left(\tau_{xz}\gamma_{xz}\right)dAdx}}$$

(7b) $$K=\frac{1}{2}\int_{0}^{L}\int_{A}\left(\dot{\bar{U}}+\dot{\bar{W}}\right)\left(\dot{\bar{U}}+\dot{\bar{W}}\right)dAdx=\frac{1}{2}\int_{0}^{L}\int_{A}\left(\rho \left(x\right){\dot{w}}^{2}+\rho \left(x\right){\left(z\dot{\theta }\right)}^{2}\right)dAdx=\frac{1}{2}\int_{0}^{L}\left(\rho \left(x\right){\dot{w}}^{2}\left(\int_{A}dA\right)+\rho \left(x\right){\dot{\theta }}^{2}\left(\int_{A}z^{2}dA\right)\right)=\frac{1}{2}\int_{0}^{L}\left(\left(\rho \left(x\right){A\left(x\right)\dot{w}}^{2}+\rho \left(x\right){I\left(x\right)\dot{\theta }}^{2}\right)\right)dx$$

(7c) $$W=\frac{1}{2}\int_{0}^{L}P_{0}{\left(\frac{\partial w}{\partial x}\right)}^{2}dx$$

Here, the superposed dot (.) represents differentiation in respect to time, *L* is the length of the beam, and *A* is the transverse area defined as a function of longitudinal coordinate owing to the axial variation of cross-sectional area. $U_{b}$ and $U_{s}$ are bending and shear strain energy, respectively. Hamilton's principle is used to drive motion equation as [71]:

(8) $$\int_{t_{1}}^{t_{2}}\left(\delta U-\delta K-\delta W\right)dt=0$$

where, δU, δK, and δW are the virtual variation of strain energy, kinetic energy, and external force work, and for each element can be written by substituting Eqs. (5) and (6) into Eq. (7) as:

(9a) $$\delta U=\delta U_{b}+\delta U_{s}$$

(9b) $$\delta U_{b}=\int_{L_{e}}\int_{A}E\left(x\right)\left(-z\frac{\partial \theta }{\partial x}\right)\left(-z\frac{\partial \delta \theta }{\partial x}\right)dAdx=\int_{L_{e}}E\left(x\right)\left(\frac{\partial \theta }{\partial x}\right)\left(\frac{\partial \delta \theta }{\partial x}\right)\left(\int_{A}Z^{2}dA\right)dx=\int_{L_{e}}E\left(x\right)I\left(x\right)\left(\frac{\partial \delta \theta }{\partial x}\right)\left(\frac{\partial \theta }{\partial x}\right)dx$$

(9c) $$\delta U_{s}=\int_{L_{e}}\int_{A}G\left(x\right)\kappa \left(\frac{\partial w}{\partial x}-\theta \right)\left(\frac{\partial \delta w}{\partial x}-\delta \theta \right)dAdx=\int_{L_{e}}G\left(x\right)\kappa \left(\frac{\partial w}{\partial x}-\theta \right)\left(\frac{\partial \delta w}{\partial x}-\delta \theta \right)\left(\int_{A}dA\right)dx=\int_{L_{e}}G\left(x\right)A\left(x\right)\kappa \left(\frac{\partial w}{\partial x}\times \frac{\partial \delta w}{\partial x}-\frac{\partial w}{\partial x}\times \delta \theta -\theta \times \frac{\partial \delta w}{\partial x}+\theta \times \delta \theta \right)dx$$

(9d) $$\delta K=-\int_{L_{e}}\left(\rho \left(x\right)A\left(x\right)\ddot{w}\delta w+\rho \left(x\right)I\left(x\right)\ddot{\theta }\delta \theta \right)dx$$

(9e) $$\delta W=\int_{L_{e}}P_{0}\left(\frac{\partial w}{\partial x}\right)\left(\frac{\partial \delta w}{\partial x}\right)dx$$

Where ρ is the mass density, and *I* is the moment of inertia; both are the function of longitudinal coordinate due to the axial variation of material properties and cross-sectional area. The general harmonic solution can be written as:

(10) $$\left\{R\left(x,t\right)\right\}=\left\{\bar{R}\right\}e^{i\omega t}$$

The natural frequency is shown by ω and $\left\{\bar{R}\right\}={\left\{w,\theta \right\}}^{T}$ is the response amplitude vector. Geometry parameterization (*x*), transverse displacement (*w*), and the rotation of cross-section (θ) are stated in the form of B-spline basis functions as:

(11a) $$x\left(\xi \right)=\sum_{i=0}^{n}N_{i,p}^{x}\left(\xi \right){{\hat{P}}_{i}}^{x}$$

(11b) $$w\left(\xi ,t\right)=\left(\sum_{i=0}^{n}N_{i,p}^{w}\left(\xi \right){Ρ}_{i}^{w}\right)e^{i\omega t}=\left[N_{0,p}^{w}\left(\xi \right)\cdots N_{n,p}^{w}\left(\xi \right)\;0\cdots 0\right]\underset{\hat{Ρ}}{\underbrace{\left[\begin{array}{c} \begin{array}{c} {Ρ}_{0}^{w}\\ \vdots \\ {Ρ}_{n}^{w} \end{array}\}{Ρ}^{w}\\ \begin{array}{c} {Ρ}_{0}^{\theta }\\ \vdots \\ {Ρ}_{n}^{\theta } \end{array}\}{Ρ}^{\theta } \end{array}\right]}}e^{i\omega t}=\left[N^{w}\right]\hat{Ρ}e^{i\omega t}$$

(11c) $$\theta \left(\xi ,t\right)=\left(\sum_{i=0}^{n}N_{i,p}^{\theta }\left(\xi \right){Ρ}_{i}^{\theta }\right)e^{i\omega t}=\left[0\cdots 0\;N_{0,p}^{\theta }\left(\xi \right)\cdots N_{n,p}^{\theta }\left(\xi \right)\right]\underset{\hat{Ρ}}{\underbrace{\left[\begin{array}{c} \begin{array}{c} {Ρ}_{0}^{w}\\ \vdots \\ {Ρ}_{n}^{w} \end{array}\}{Ρ}^{w}\\ \begin{array}{c} {Ρ}_{0}^{\theta }\\ \vdots \\ {Ρ}_{n}^{\theta } \end{array}\}{Ρ}^{\theta } \end{array}\right]}}e^{i\omega t}=\left[N^{\theta }\right]\hat{Ρ}e^{i\omega t}$$

Where ${\hat{P}}_{i}^{x}$, ${\hat{P}}_{i}^{w}$ and ${\hat{P}}_{i}^{\theta }$ are the *i*th control points of physical coordinate, deflection, and rotation, respectively. Based on Eq. (11), the virtual variation of transverse displacement (*w*) and the rotation of cross-section (θ) and variation of these parameters in respect to physical coordinate (*x*) can be written as:

(12a) $$\delta w=\left(\sum_{i=0}^{n}N_{i,p}^{w}\left(\xi \right)\delta {Ρ}_{i}^{w}\right)e^{i\omega t}=\left[N_{0,p}^{w}\left(\xi \right)\cdots N_{n,p}^{w}\left(\xi \right)\;0\cdots 0\right]\delta \hat{Ρ}e^{i\omega t}=\left[N^{w}\right]\delta \hat{Ρ}e^{i\omega t}$$

(12b) $$\delta \theta =\left(\sum_{i=0}^{n}N_{i,p}^{\theta }\left(\xi \right)\delta {Ρ}_{i}^{\theta }\right)e^{i\omega t}=\left[0\cdots 0\;N_{0,p}^{\theta }\left(\xi \right)\cdots N_{n,p}^{\theta }\left(\xi \right)\right]\delta \hat{Ρ}e^{i\omega t}=\left[N^{\theta }\right]\delta \hat{Ρ}e^{i\omega t}$$

(12c) $$\frac{\partial w}{\partial x}=\left[\frac{\partial N_{0,p}^{w}}{\partial x},\ldots ,\frac{\partial N_{n,p}^{w}}{\partial x},0,\ldots ,0\right]\left[\hat{Ρ}\right]e^{i\omega t}=\left[\frac{\partial N^{w}}{\partial x}\right]\left[\hat{Ρ}\right]e^{i\omega t}$$

(12d) $$\frac{\partial \delta w}{\partial x}=\left[\frac{\partial N_{0,p}^{w}}{\partial x},\ldots ,\frac{\partial N_{n,p}^{w}}{\partial x},0,\ldots ,0\right]\left[\delta \hat{Ρ}\right]e^{i\omega t}=\left[\frac{\partial N^{w}}{\partial x}\right]\left[\delta \hat{Ρ}\right]e^{i\omega t}$$

(12e) $$\frac{\partial \theta }{\partial x}=\left[0,\ldots ,0,\frac{\partial N_{0,p}^{\theta }}{\partial x},\ldots ,\frac{\partial N_{n,p}^{\theta }}{\partial x}\right]\left[\hat{Ρ}\right]e^{i\omega t}=\left[\frac{\partial N^{\theta }}{\partial x}\right]\left[\hat{Ρ}\right]e^{i\omega t}$$

(12f) $$\frac{\partial \delta \theta }{\partial x}=\left[0,\ldots ,0,\frac{\partial N_{0,p}^{\theta }}{\partial x},\ldots ,\frac{\partial N_{n,p}^{\theta }}{\partial x}\right]\left[\delta \hat{Ρ}\right]e^{i\omega t}=\left[\frac{\partial N^{\theta }}{\partial x}\right]\left[\delta \hat{Ρ}\right]e^{i\omega t}$$

Also based on Eq. (11), $\ddot{w}$ and $\ddot{\theta }$ can be written as

(13a) $$\ddot{w}=-\omega^{2}\left(\sum_{i=0}^{n}N_{i,p}^{w}\left(\xi \right){Ρ}_{i}^{w}\right)e^{i\omega t}=-\omega^{2}\left[N_{0,p}^{w}\left(\xi \right)\cdots N_{n,p}^{w}\left(\xi \right)\;0\cdots 0\right]\hat{Ρ}e^{i\omega t}=-\omega^{2}\left[N^{w}\right]\hat{Ρ}e^{i\omega t}$$

(13b) $$\ddot{\theta }=-\omega^{2}\left(\sum_{i=0}^{n}N_{i,p}^{\theta }\left(\xi \right){Ρ}_{i}^{\theta }\right)e^{i\omega t}=-\omega^{2}\left[0\cdots 0\;N_{0,p}^{\theta }\left(\xi \right)\cdots N_{n,p}^{\theta }\left(\xi \right)\right]\hat{Ρ}e^{i\omega t}=-\omega^{2}\left[N^{\theta }\right]\hat{Ρ}e^{i\omega t}$$

By substituting Eqs. (11)–(13) into Eq. (9), the bending (*k*_*b*_), shear (*k*_*s*_), geometric (*k*_*g*_) stiffness matrices and, the mass matrix (*m*) can be computed for an element as follow:

(14a) $$\delta U_{b}=\delta {\hat{Ρ}}^{T}k_{b}\hat{Ρ}e^{2i\omega t}\;k_{b}=\int_{L_{e}}E\left(x\right)I\left(x\right){\left[\frac{\partial N^{\theta }}{\partial x}\right]}^{T}\left[\frac{\partial N^{\theta }}{\partial x}\right]dx$$

(14b) $$\delta U_{s}={\left[\delta \hat{Ρ}\right]}^{T}k_{s}\left[\hat{Ρ}\right]e^{2i\omega t}k_{s}=\int_{L_{e}}G\left(x\right)A\left(x\right)\kappa \left({\left[\frac{\partial N^{w}}{\partial x}\right]}^{T}\left[\frac{\partial N^{w}}{\partial x}\right]-{\left[N^{\theta }\right]}^{T}\left[\frac{\partial N^{w}}{\partial x}\right]-{\left[\frac{\partial N^{w}}{\partial x}\right]}^{T}\left[N^{\theta }\right]+{\left[N^{\theta }\right]}^{T}\left[N^{\theta }\right]\right)dx$$

(14c) $$\begin{array}{l} \delta K=-\int_{l_{e}}-\omega^{2}\left(\rho \left(x\right)A\left(x\right){\left[\delta \hat{Ρ}\right]}^{T}{\left[N^{w}\right]}^{T}\left[N^{w}\right]\left[\delta \hat{Ρ}\right]+\rho \left(x\right)I\left(x\right){\left[\delta \hat{Ρ}\right]}^{T}{\left[N^{\theta }\right]}^{T}\left[N^{\theta }\right]\left[\delta \hat{Ρ}\right]\right)\;dx\;e^{2i\omega t}\;=\omega^{2}{\left[\delta \hat{Ρ}\right]}^{T}\;m\left[\delta \hat{Ρ}\right]e^{2i\omega t}\\ m=\int_{L_{e}}\left(\rho \left(x\right)A\left(x\right){\left[N^{w}\right]}^{T}\left[N^{w}\right]+\rho \left(x\right)I\left(x\right){\left[N^{\theta }\right]}^{T}\left[N^{\theta }\right]\right)dx \end{array}$$

(14d) $$\delta W={\left[\delta \hat{Ρ}\right]}^{T}k_{G}\left[\hat{Ρ}\right]e^{2i\omega t}\;k_{G}={\int_{L_{e}}P_{0}\left[\frac{\partial N^{w}}{\partial x}\right]}^{T}\left[\frac{\partial N^{w}}{\partial x}\right]dx$$

By differentiating from Eq. (11a), the Jacobian of transformation from the physical domain to the parametric domain can be computed as:

(15) $$dx\left(\xi \right)=\sum_{i=0}^{n}N_{i,p}^{\prime }\left(\xi \right){Ρ}_{i}^{x}d\xi \to J=\frac{dx}{d\xi }=\sum_{i=0}^{n}N_{i,p}^{\prime }\left(\xi \right){Ρ}_{i}^{x}$$

By substituting Eq. (15) into Eq. (14), the bending (*k*_*b*_), shear (*k*_*s*_), geometric (*k*_*g*_) stiffness matrices and, the mass matrix (*m*) can be computed for an element in the parametric domain as follows:

(16a) $$k_{b}=\int_{\xi_{i}}^{\xi_{i+1}}E\left(\xi \right)I\left(\xi \right){\left[\frac{\partial N^{w}}{\partial \xi }\right]}^{T}\left[\frac{\partial N^{w}}{\partial \xi }\right]J^{-1}d\xi$$

(16b) $$k_{s}=\int_{\xi_{i}}^{\xi_{i+1}}\kappa A\left(\xi \right)G\left(\xi \right){\left[\frac{\partial N^{w}}{\partial \xi }\times J^{-1}-N^{\theta }\right]}^{T}\left[\frac{\partial N^{w}}{\partial \xi }\times J^{-1}-N^{\theta }\right]Jd\xi$$

(16c) $$m=\int_{\xi_{i}}^{\xi_{i+1}}\left(\rho \left(\xi \right)A\left(\xi \right){\left[N^{w}\right]}^{T}{\left[N\right]}^{w}+\rho \left(\xi \right)I\left(\xi \right){\left[N^{\theta }\right]}^{T}{\left[N\right]}^{\theta }\right)Jd\xi$$

(16d) $$k_{G}=\int_{\xi_{i}}^{\xi_{i+1}}P_{0}{\left[\frac{\partial N^{w}}{\partial \xi }\right]}^{T}\left[\frac{\partial N^{w}}{\partial \xi }\right]J^{-1}d\xi$$

**Fig. 3:**
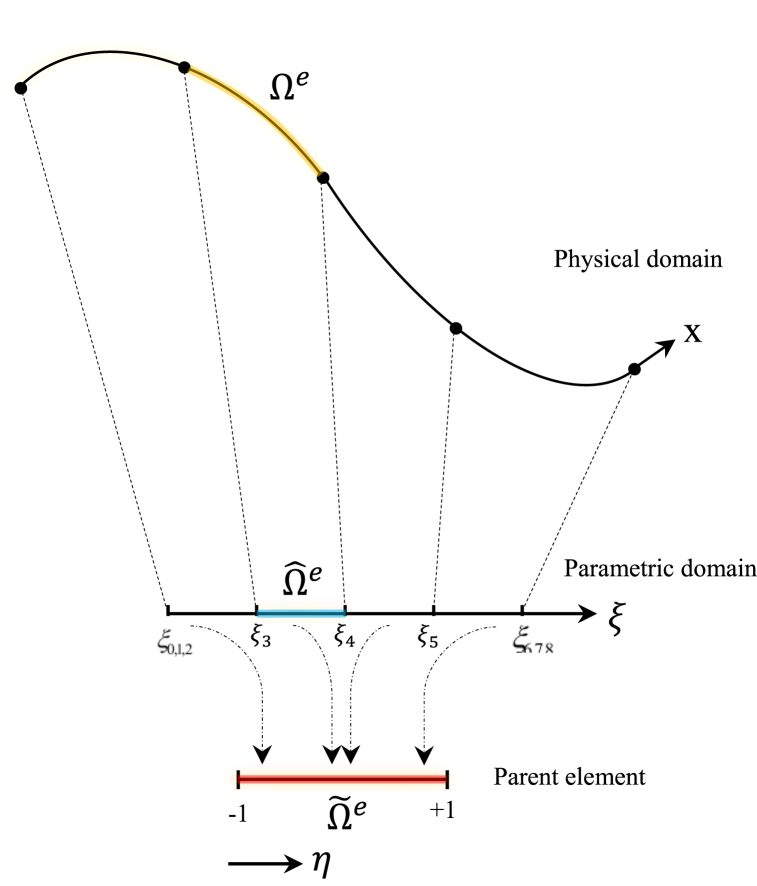
Physical, parametric, and parent elements for Gauss quadrature and knot vector Ξ={0,0,0,0.25,0.5,0.75,1,1,1}.

To compute the numerical integration over the parametric domain, the Gauss-Legendre quadrature needs at least $n_{q}=\lceil \left(p+1\right)/2\rceil$ Gauss points to the integrate polynomials of degree *p*, which is applied throughout this study. This notation ⌈D⌉ means the closest integer larger than *D*. Elements in the physical, parametric, and parent domain are depicted in Fig. 3. To compute the value of the quadrature point in the parametric domain $\xi_{q}\in {\overset{\;}{\Omega }}^{e}|{\overset{\;}{\Omega }}^{e}=\left[\xi_{i},\xi_{i+1}\right]$ from the quadrature point in the parent element $\eta_{q}\in {\tilde{\Omega }}^{e}|{\tilde{\Omega }}^{e}=\left[-1,1\right]$, the following mapping equation is applied.

(17) $$\xi_{q}=\frac{\eta_{q}+1}{2}\left(\xi_{i+1}-\xi_{i}\right)+\xi_{i}$$

It should be pointed out that the assembly procedure in IGA and finite-element approaches are the same. After the assembling process, the global bending stiffness (*K*_*b*_), shear stiffness (*K*_*s*_), geometric stiffness (*K*_*g*_) matrices, and mass matrix (*M*) are acquired from Eq. (16). After imposing boundary conditions on these matrices, the subsequent eigenvalue equation should be solved to investigate free vibration and buckling of AFG tapered beam, respectively [23].

Free vibration analysis: $\left(K_{b}+K_{s}-\omega^{2}M\right)\Psi =0$

Buckling analysis: $\left(K_{b}+K_{s}-\lambda K_{G}\right)\Psi =0$

Here Ψ is the eigenvector and λ is the eigenvalue applied in computing the critical load $P_{cr}=\lambda P_{0}$, where *P*_*0*_ is the axial load which is required for computing the geometric stiffness matrix.

By using the IGA rule, a computer program in Matlab is developed in which the natural frequencies, mode shapes, and critical loads are derived. Multiple figures and tables depict the impacts of various parameters. For more details on the isogeometric method and its application, the interested reader can refer to Ref. [74].

### 2.3 System configurations

In this study, three different cases of the cross-sectional profile are applied as the geometry of the beam:

Case 1 Linearly tapering height and constant breadth;

$$A\left(x\right)=H_{0}\times B_{0}\left(1-C_{h}\frac{x}{L}\right)\;I\left(x\right)=\frac{B_{0}\times H_{0}^{3}}{12}{\left(1-C_{h}\frac{x}{L}\right)}^{3}$$

Case 2 linearly tapering height and breadth;

$$A\left(x\right)=H_{0}\times B_{0}\left(1-C_{b}\frac{x}{L}\right)\left(1-C_{h}\frac{x}{L}\right)\;I\left(x\right)=\frac{B_{0}\times H_{0}^{3}}{12}\left(1-C_{b}\frac{x}{L}\right){\left(1-C_{h}\frac{x}{L}\right)}^{3}$$

Case 3 linearly tapering breadth and constant height;

$$A\left(x\right)=H_{0}\times B_{0}\left(1-C_{b}\frac{x}{L}\right)\;I\left(x\right)=\frac{B_{0}\times H_{0}^{3}}{12}\left(1-C_{b}\frac{x}{L}\right)$$

**Fig. 4:**
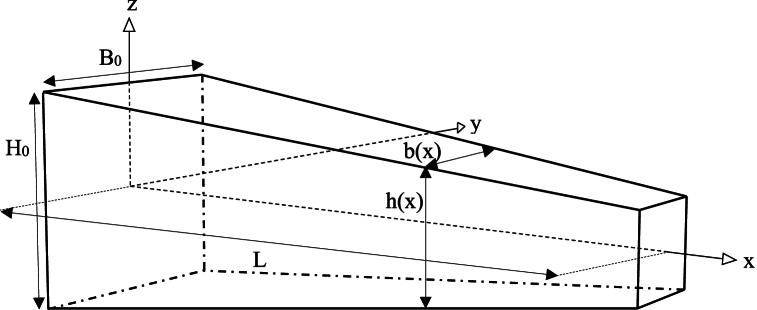
Configuration of a tapered beam.

**Fig. 5:**
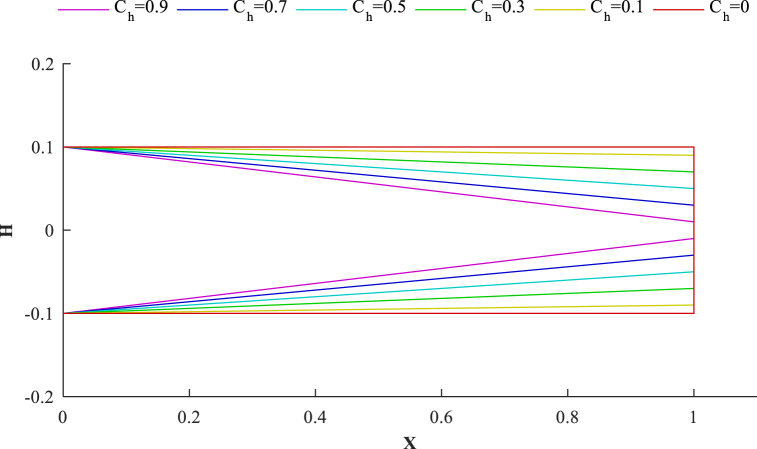
Variation of lateral surface for different values of *C*_*h*_ (case 1, *h/L* = 0.2).

C_h_ and C_b_ are the height, and the breadth taper ratio, which varies in 0≤C<1 range, and H_0_ and B_0_ are the height and breadth of the left end cross-section, as shown in Fig. 4. As an example, for case 1, the lateral surface of a beam is depicted for various values of C_h_ in Fig. 5.

The effective material properties change in the beam axis direction and are computed according to the following power-law relation.

$$P_{eff}\left(x\right)=\left(P_{R}-P_{L}\right){\left(\frac{x}{L}\right)}^{n}+P_{R}$$

**Fig. 6:**
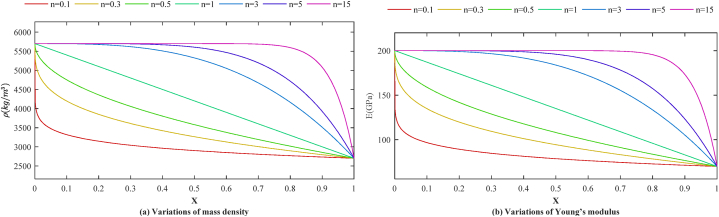
(a) Variation of mass density and (b) Young's modulus for different values of the non-homogeneity parameters.

Where P_R_ and P_L_ are the material properties like *E*, ρ, and G at the right and left end cross-section, and n is the material non-homogeneity parameter. The change of mass density and Young's modulus in the beam axis direction with respect to the different values of n are illustrated for the unit length $ZrO_{2}/Al$ AFG beam in Fig. 6. The properties of Aluminum and Zirconia are as follows:

Aluminum (Al): $\rho =2702\;\text{kg}/m^{3},E=70\;\text{GPa}$

Zirconia (ZrO_2_): $\rho =5700\;\text{kg}/m^{3},E=200\;\text{GPa}$

From Fig. 6, one can realize that for n>3, and n<1/3 the percentage of one of the constituent materials is highly increased; thus, it is suggested by Nakamura et al. [75] that n vary in (1/3≤n≤3) range.

## 3 Numerical results

To specify the competency and precision of the isogeometric method in the free vibration and buckling of AFG tapered Timoshenko beams, some numerical examples are presented. In what follows; first, the shear locking phenomenon in slender beams and the convergence rate of the IGA method are presented; next, the effects of several parameters like the non-homogeneity parameter and taper ratio are investigated in the free vibration and buckling of a beam with various boundary conditions.

To specify the boundary conditions, the symbols *C*, *F,* and *S* are used for clamped, free, and simply supported boundary conditions, respectively. For convenience, the following dimensionless parameters are considered:

Mass density ratio: $\rho_{ratio}=\frac{\rho_{R}}{\rho_{L}}$

Young's modulus ratio: $E_{ratio}=\frac{E_{R}}{E_{L}}$

Dimensionless critical load: $\bar{P}=\frac{P_{cr}L^{2}}{E_{L}I_{L}}$

*i*th Dimensionless natural frequency: ${\bar{\omega }}_{i}=\omega_{i}\sqrt{\frac{\rho_{L}A_{L}L^{4}}{E_{L}I_{L}}}$

where the subscript *L* and *R* indicate the value of each parameter at the left and right end cross-section, respectively. In all the following examples, it is supposed that the shear correction factor and the Poisson's ratio are k=5/6 and, ν=0.3 respectively.

### 3.1 Shear locking phenomenon and convergence study

**Fig. 7:**
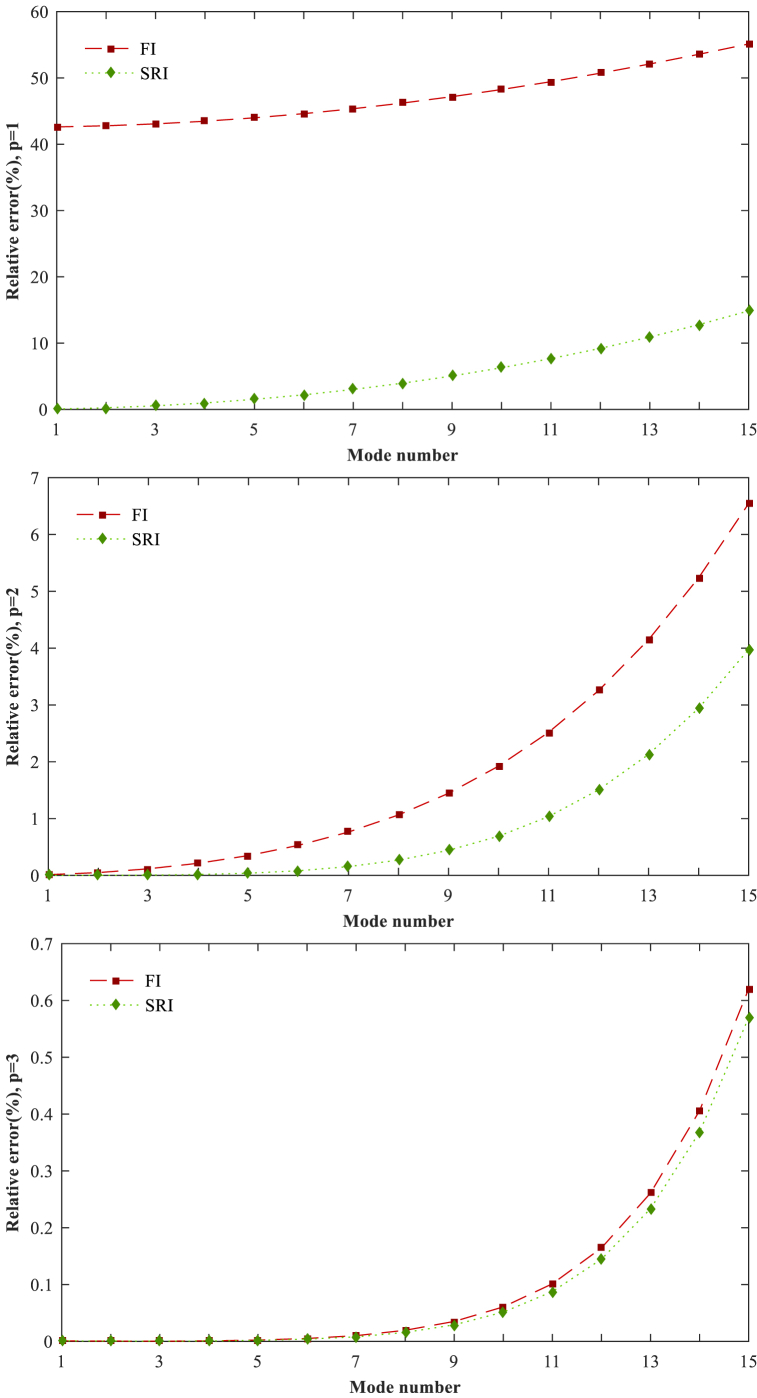
The relative errors (%) of full integration (FI) and selective reduced integration (SRI) with different orders (*p* = 1, 2, 3) for different mode numbers.

Two methods are adopted to assess the number of Gauss integration points on the shear-locking phenomenon. The first method is full integration (FI), where the bending and shear stiffness matrices (Kb & Ks) are completely integrated. The second method is selectively reduced integration (SRI) which applies full integration for the bending stiffness matrix and reduced integration for the shear stiffness matrix. It is important to note that SRI has lower computational costs in comparison with FI and is applied easily. As a numerical example, a unit-length S-S homogeneous beam is studied with the different order of basis function (*p*) and the fixed number of the isogeometric elements (*N*_*el*_ = 32). For both integration methods, the results are presented in Table 1. The relative error percentages are illustrated in Fig. 7 and defined as:

$$\text{Relative}\;\text{error}\left(\%\right)=\left|\frac{{\bar{\Omega }}_{i}-{{\bar{\Omega }}_{i}}^{\left\{P\right\}}}{{{\bar{\Omega }}_{i}}^{\left\{P\right\}}}\right|\times 100$$

Where ${\bar{\Omega }}_{i}$ and ${{\bar{\Omega }}_{i}}^{\left\{P\right\}}$ are the square root of *i*th natural frequencies in this study and the Pseudospectral method [76], respectively. It is observed that the isogeometric method can suffer from the shear-locking phenomenon when the basic functions with lower-order (*p* = 1 and 2) are applied. The SRI obtains better results than FI and can efficiently mitigate the shear-locking effect, especially when the lower order of basis function is applied. Thus, in this study, the results are computed through the SRI method.

**Table 1:** Effect of different integration methods on the first fifteen ${\bar{\Omega }}_{i}=\sqrt{{\bar{\omega }}_{i}}$ of S-S homogeneous beam (*h/L* = 0.01, *N*_*el*_ = *32*).

Mode number	FI method			SRI method			Pseudospectral method
	P = 1	P = 2	P = 3	P = 1	P = 2	P = 3	
1	4.48	3.1417	3.1413	3.1432	3.1413	3.1413	3.1413
2	8.9685	6.2842	6.2811	6.2962	6.2811	6.2811	6.2811
3	13.474	9.4286	9.4176	9.4689	9.4181	9.4176	9.4176
4	18.005	12.5763	12.5495	12.6712	12.5514	12.5495	12.5494
5	22.5699	15.7297	15.6753	15.9135	15.681	15.6752	15.6749
6	27.1772	18.8921	18.7936	19.2064	18.8081	18.7934	18.7926
7	31.835	22.0683	21.9033	22.561	21.935	21.9028	21.9011
8	36.5516	25.2648	25.0037	25.9889	25.0662	25.0028	24.9988
9	41.3346	28.4903	28.0944	29.5023	28.2085	28.0927	28.0845
10	46.1916	31.7559	31.1757	33.1141	31.3716	31.1728	31.1568
11	51.1293	35.076	34.2492	36.8379	34.5688	34.2445	34.2145
12	56.1538	38.4679	37.3178	40.6881	37.8174	37.3104	37.2565
13	61.2699	41.953	40.3867	44.6802	41.1395	40.3756	40.2815
14	66.4812	45.556	43.4644	48.8301	44.5621	43.4481	43.2886
15	71.7889	49.3053	46.564	53.1548	48.1172	46.5406	46.2769

*Note*. Pseudospectral method: Ref [76].

To define the required order and number of isogeometric elements to obtain acceptable and converged results, a tapered (*C*_*h*_ = 0.5, *C*_*b*_ = 0) $ZrO_{2}/Al$ AFG (*n* = 2) beam is of concern here. In Table 2, ${\bar{\omega }}_{i}\left(i=1,2,3,4\right)$ are computed for different orders and numbers of elements in the S-S beam and compared with those obtained with DTEM and DQEL methods [77].

**Table 2:** The first four ${\bar{\omega }}_{i}=\omega_{i}L^{2}\sqrt{\frac{\rho A}{EI}}$ of S-S AFG (*n* = 2) tapered (*C*_*h*_ = 0.5, *C*_*b*_ = 0) beam (*h/L* = $\sqrt{0.12}$) for different orders (*p*) and different number of elements (*N*_*el*_).

P	N_el_	${\bar{\omega }}_{1}$	${\bar{\omega }}_{2}$	${\bar{\omega }}_{3}$	${\bar{\omega }}_{4}$	P	N_el_	${\bar{\omega }}_{1}$	${\bar{\omega }}_{2}$	${\bar{\omega }}_{3}$	${\bar{\omega }}_{4}$
1	1					4	1	5.9872	21.6069	41.3909	
	2	7.5030					2	5.9890	20.9479	40.7535	65.2257
	4	6.3679	25.7787	55.3305			4	5.9846	20.8139	38.2063	57.5876
	8	6.0838	21.9983	42.3711	65.0958		8	5.9845	20.8125	38.1221	56.2314
	16	6.0097	21.1054	39.1556	58.6151		16	5.9845	20.8125	38.1219	56.2264
	32	5.9908	20.8855	38.3783	56.8172		32	5.9845	20.8125	38.1219	56.2263
	64	5.9861	20.8307	38.1859	56.3736		64	5.9845	20.8125	38.1219	56.2263
	128	5.9849	20.8170	38.1379	56.2631		128	5.9845	20.8125	38.1219	56.2263
2	1	5.8187				5	1	5.9852	20.7384	40.8496	64.8813
	2	6.0673	23.5418				2	5.9852	20.8161	38.5475	62.1346
	4	5.9993	21.1730	41.1154	64.0514		4	5.9845	20.8125	38.1379	56.5640
	8	5.9855	20.8309	38.2673	56.9083		8	5.9845	20.8125	38.1219	56.2267
	16	5.9845	20.8134	38.1288	56.2556		16	5.9845	20.8125	38.1219	56.2263
	32	5.9845	20.8125	38.1223	56.2279		32	5.9845	20.8125	38.1219	56.2263
	64	5.9845	20.8125	38.1219	56.2264		64	5.9845	20.8125	38.1219	56.2263
	128	5.9845	20.8125	38.1219	56.2264		128	5.9845	20.8125	38.1219	56.2263
3	1	6.0749	21.0531			6	1	5.9847	20.8007	37.9873	62.4642
	2	6.0109	21.6264	44.2141			2	5.9846	20.8126	38.1346	57.3033
	4	5.9858	20.8447	38.8419	62.6171		4	5.9845	20.8125	38.1225	56.3140
	8	5.9845	20.8128	38.1276	56.2824		8	5.9845	20.8125	38.1219	56.2264
	16	5.9845	20.8125	38.1219	56.2269		16	5.9845	20.8125	38.1219	56.2263
	32	5.9845	20.8125	38.1219	56.2264		32	5.9845	20.8125	38.1219	56.2263
	64	5.9845	20.8125	38.1219	56.2263		64	5.9845	20.8125	38.1219	56.2263
	128	5.9845	20.8125	38.1219	56.2263		128	5.9845	20.8125	38.1219	56.2263
DTEM		5.9845	20.8125	38.1219	56.2263						
DQEL		5.9845	20.8123	38.1250	56.2256						

*Note.* DTEM & DQEL: Ref [77].

It can be seen that the IGA results are in accord with the DQEM and DQEL by using 64 cubic or 16 quintic elements etc. Thus, in the numerical examples, 64 cubic elements will be applied unless stated otherwise.

### 3.2 Free vibration

The free vibration of AFG tapered beams is studied for different boundary conditions [78]. Moreover, the effect of the non-homogeneity parameters, taper ratio, and *E* ratio are investigated [79]. Here, it is assumed that the AFG beams are made of *ZrO*_2_ at the left end and *Al* at the right end, and the thickness-to-length ratio is taken as *h/L* = $\sqrt{0.12}$, unless otherwise stated.

#### 3.2.1 Validation

Inorder to ensure reliabilityand verify the present method, the results are compered with some other related reference [77].The ${\bar{\omega }}_{i}\left(i=1,2,3,4\right)$ of the AFG uniform beams are tabulated in Table 3 for different non-homogeneity parameters, thickness-to-length ratios, and boundary conditions. The IGA results are in accord with the results of other methods [77]. The ratio of the ${\bar{\omega }}_{i}\left(i=1,2,3,4\right)$ in the AFG beams with different material non-homogeneity parameters n (0.3≤n≤3) to those with n = 0.3 (${\bar{\omega }}_{0.3}$) is presented for various boundary conditions. As listed in Table 3, an excellent agreement is observed due to this comparison.

**Table 3:** Comparison between the present work and related results of Ref. [77] for ${\bar{\omega }}_{i}\left(i=1,2,3,4\right)$

h/L	n	Method	C-F				S-S				C-C			
			${\bar{\omega }}_{1}$	${\bar{\omega }}_{2}$	${\bar{\omega }}_{3}$	${\bar{\omega }}_{4}$	${\bar{\omega }}_{1}$	${\bar{\omega }}_{2}$	${\bar{\omega }}_{3}$	${\bar{\omega }}_{4}$	${\bar{\omega }}_{1}$	${\bar{\omega }}_{2}$	${\bar{\omega }}_{3}$	${\bar{\omega }}_{4}$
0.12^0.5^	1	Present	3.8963	15.0505	30.9409	46.3782	7.8459	23.9406	41.6789	53.7111	12.9065	26.7597	43.0417	58.5205
		DTEM	3.8963	15.0505	30.9409	46.3782	7.8459	23.9406	41.6789	53.7111	12.9065	26.7597	43.0417	58.5205
		DQEL	3.8963	15.0504	30.9405	46.3776	7.8459	23.9404	41.6783	53.7100	12.9064	26.7593	43.0410	58.5196
	2	Present	3.8828	15.2591	31.6180	47.6349	7.9873	24.2521	42.3024	54.8771	12.6873	26.6416	43.3694	59.2779
		DTEM	3.8828	15.2590	31.6180	47.6349	7.9873	24.2521	42.3024	54.8771	12.6873	26.6416	43.3694	59.2779
		DQEL	3.8828	15.2589	31.6177	47.6343	7.9872	24.2519	42.3018	54.8760	12.6871	26.6413	43.3688	59.2771
	3	Present	3.7957	15.2936	31.9962	48.3335	8.0645	24.3532	42.5010	55.3881	12.6029	26.5810	43.4958	59.5726
		DTEM	3.7957	15.2936	31.9962	48.3335	8.0645	24.3532	42.5010	55.3881	12.6030	26.5809	43.4958	59.5726
		DQEL	3.7956	15.2935	31.9958	48.3328	8.0644	24.3529	42.5004	55.3870	12.6028	26.5806	43.4952	59.5717

It is noteworthy that for *ZrO*_2_*/Al* AFG beams, a rise in *n* results in a heavier and stiffer beam, as illustrated in Fig. 6. Due to the sensitivity of natural frequencies to the stiffness and mass of the beam, the variation of the natural frequencies concerning *n* is unpredictable.

#### 3.2.2 Effects of taper ratio

In this part, the numerical examples show the effects of the taper ratio on the natural frequencies of AFG tapered beams for various boundary conditions. The ${\bar{\omega }}_{i}\left(i=1,2,3,4\right)$ of C-F, S-S, and C-C AFG tapered beams are tabulated in Table 4, Table 5, Table 6, respectively, for a constant non-homogeneity parameter (*n* = 2) and different tapering cases and taper ratios. The results obtained from the IGA method indicate an excellent consistency with DTEM [77] and FEM [23]. As mentioned before, in case 1, the height taper ratio is variable and the breadth taper ratio is set to *C*_*b*_ = 0, the height and breadth taper ratios vary with the same value (*C*_*h*_ = *C*_*b*_) in case 2, and the breadth taper ratio is variable while the height taper ratio is set to *C*_*h*_ = 0 in case 3.

**Table 4:** Influence of taper ratios on in AFG tapered beam (n = 2); C-F.

Taper ratio	Method	Case 1				Case 2				Case 3			
		${\bar{\omega }}_{1}$	${\bar{\omega }}_{2}$	${\bar{\omega }}_{3}$	${\bar{\omega }}_{4}$	${\bar{\omega }}_{1}$	${\bar{\omega }}_{2}$	${\bar{\omega }}_{3}$	${\bar{\omega }}_{4}$	${\bar{\omega }}_{1}$	${\bar{\omega }}_{2}$	${\bar{\omega }}_{3}$	${\bar{\omega }}_{4}$
0	Present	3.8828	15.2591	31.6180	47.6349	3.8828	15.2591	31.6180	47.6349	3.8828	15.2591	31.6180	47.6349
	DTEM	3.8828	15.2590	31.6180	47.6349	3.8828	15.2590	31.6180	47.6349				
0.1	Present	3.9358	15.1533	31.2239	47.5836	4.0491	15.3084	31.3393	47.6898	3.9959	15.4185	31.7342	47.7475
	DTEM	3.9358	15.1533	31.2239	47.5836	4.0491	15.3084	31.3393	47.6898				
	FEM	3.9359	15.1577	31.2638	47.7164	4.0492	15.3129	31.3795	47.8232				
0.2	Present	3.9954	15.0201	30.7703	47.3011	4.2381	15.3392	31.0149	47.5184	4.1243	15.5961	31.8657	47.8785
	DTEM	3.9954	15.0201	30.7703	47.3011	4.2381	15.3392	31.0149	47.5184				
	FEM	3.9956	15.0247	30.8092	47.4362	4.2384	15.3441	31.0546	47.6550				
0.3	Present	4.0637	14.8563	30.2481	46.8133	4.4560	15.3523	30.6404	47.1533	4.2720	15.7966	32.0174	48.0341
	DTEM	4.0637	14.8562	30.2481	46.8133	4.4560	15.3523	30.6404	47.1532				
	FEM	4.0640	14.8611	30.2860	46.9481	4.4565	15.3579	30.6798	47.2907				
0.4	Present	4.1434	14.6582	29.6445	46.1282	4.7112	15.3505	30.2097	46.6112	4.4444	16.0272	32.1964	48.2235
	DTEM	4.1434	14.6582	29.6445	46.1282	4.7112	15.3505	30.2097	46.6112				
	FEM	4.1438	14.6636	29.6818	46.2610	4.7121	15.3573	30.2496	46.7483				
0.5	Present	4.2385	14.4219	28.9420	45.2337	5.0164	15.3401	29.7157	45.8922	4.6491	16.2987	32.4139	48.4613
	DTEM	4.2385	14.4219	28.9420	45.2337	5.0164	15.3401	29.7157	45.8922				
	FEM	4.2393	14.4282	28.9791	45.3634	5.0178	15.3487	29.7571	46.0287				
0.6	Present	4.3560	14.1434	28.1153	44.0936	5.3908	15.3350	29.1512	44.9797	4.8979	16.6293	32.6897	48.7718
	DTEM	4.3560	14.1434	28.1153	44.0936	5.3908	15.3350	29.1513	44.9797				
	FEM	4.3571	14.1513	28.1532	44.2201	5.3931	15.3467	29.1962	45.1172				
0.7	Present	4.5073	13.8210	27.1276	42.6380	5.8659	15.3687	28.5154	43.8406	5.2091	17.0524	33.0617	49.1999
	DTEM	4.5073	13.8210	27.1276	42.6380	5.8659	15.3687	28.5154	43.8406				
	FEM	4.5090	13.8314	27.1684	42.7630	5.8695	15.3856	28.5680	43.9835				
0.8	Present	4.7154	13.4641	25.9250	40.7369	6.4951	15.5302	27.8403	42.4322	5.6139	17.6374	33.6171	49.8401
	DTEM	4.7154	13.4641	25.9250	40.7369	6.4951	15.5302	27.8403	42.4322				
	FEM	4.7180	13.4793	25.9735	40.8666	6.5009	15.5568	27.9102	42.5941				
0.9	Present	5.0330	13.1443	24.4495	38.1378	7.3695	16.1061	27.3632	40.8137	6.1694	18.5548	34.6163	50.9638
	DTEM	5.0330	13.1443	24.4495	38.1378	7.3695	16.1061	27.3632	40.8137				
	FEM	5.0371	13.1700	24.5212	38.2968	7.3787	16.1537	27.4806	41.0442				

*Note.* DTEM: Ref [77]. FEM: Ref [23].

**Table 5:** Influence of taper ratios on ${\bar{\omega }}_{i}\left(i=1,2,3,4\right)$ in AFG tapered beam (*n* = *2*); S-S.

Taper ratio	Method	Case 1				Case 2				Case 3			
		${\bar{\omega }}_{1}$	${\bar{\omega }}_{2}$	${\bar{\omega }}_{3}$	${\bar{\omega }}_{4}$	${\bar{\omega }}_{1}$	${\bar{\omega }}_{2}$	${\bar{\omega }}_{3}$	${\bar{\omega }}_{4}$	${\bar{\omega }}_{1}$	${\bar{\omega }}_{2}$	${\bar{\omega }}_{3}$	${\bar{\omega }}_{4}$
0	Present	7.9873	24.2521	42.3024	54.8771	7.9873	24.2521	42.3024	54.8771	7.9873	24.2521	42.3024	54.8771
	DTEM	7.9873	24.2521	42.3024	54.8771	7.9873	24.2521	42.3024	54.8771				
0.3	Present	6.8952	22.4377	40.1984	58.4515	6.7736	22.4541	40.2154	58.4628	7.8870	24.2310	42.2695	55.1550
	DTEM	6.8952	22.4377	40.1984	58.4515	6.7736	22.4541	40.2154	58.4628				
	FEM	6.8975	22.4604	40.3053	58.7642	6.7764	22.4783	40.3248	58.7787				
0.6	Present	5.4495	19.8133	36.7600	54.6859	5.0642	19.9614	36.9293	54.8194	7.6520	24.1328	42.1555	55.6693
	DTEM	5.4495	19.8133	36.7600	54.6859	5.0643	19.9614	36.9293	54.8194				
	FEM	5.4540	19.8372	36.8536	54.9547	5.0709	19.9917	37.0337	55.1026				
0.9	Present	3.1846	15.3215	29.7694	45.9402	2.2924	16.2122	30.7672	46.8831	6.8541	23.4085	41.3672	56.7710
	DTEM	3.1846	15.3215	29.7694	45.9402	2.2924	16.2122	30.7672	46.8831				
	FEM	3.2016	15.3775	29.9011	46.2153	2.3193	16.2962	30.9578	47.2473				

*Note.* DTEM: Ref [77]. FEM: Ref [23].

**Table 6:** Influence of taper ratios on ${\bar{\omega }}_{i}\left(i=1,2,3,4\right)$ in AFG tapered beam (*n* = 2); C-C.

Taper ratio	Method	Case 1				Case 2				Case 3			
		${\bar{\omega }}_{1}$	${\bar{\omega }}_{2}$	${\bar{\omega }}_{3}$	${\bar{\omega }}_{4}$	${\bar{\omega }}_{1}$	${\bar{\omega }}_{2}$	${\bar{\omega }}_{3}$	${\bar{\omega }}_{4}$	${\bar{\omega }}_{1}$	${\bar{\omega }}_{2}$	${\bar{\omega }}_{3}$	${\bar{\omega }}_{4}$
0	Present	12.6873	26.6416	43.3694	59.2779	12.6873	26.6416	43.3694	59.2779	12.6873	26.6416	43.3694	59.2779
	DTEM	12.6873	26.6416	43.3694	59.2779	12.6873	26.6416	43.3694	59.2779				
0.3	Present	11.9112	25.6899	41.9520	58.7487	11.9670	25.7737	42.0199	58.8154	12.6887	26.6754	43.4064	59.3509
	DTEM	11.9112	25.6899	41.9520	58.7488	11.9670	25.7737	42.0199	58.8154				
	FEM	11.9172	25.7236	42.0759	59.0576	11.9737	25.8089	42.1461	59.1273				
0.6	Present	10.6807	23.9078	39.5223	56.1880	10.9080	24.1861	39.7385	56.3600	12.5612	26.5805	43.3598	59.3996
	DTEM	10.6807	23.9078	39.5224	56.1880	10.9080	24.1861	39.7385	56.3600				
	FEM	10.6896	23.9424	39.6375	56.4814	10.9200	24.2270	39.8636	56.6668				
0.9	Present	8.3194	19.6018	33.3779	48.7765	9.2522	20.6544	34.3131	49.5537	11.7853	25.7370	42.7172	59.0465
	DTEM	8.3194	19.6019	33.3779	48.7765	9.2522	20.6544	34.3131	49.5538				
	FEM	8.3590	19.6945	33.5615	49.1234	9.3010	20.7781	34.5540	49.9856				

*Note.* DTEM: Ref [77]. FEM: Ref [23].

**Fig. 8:**
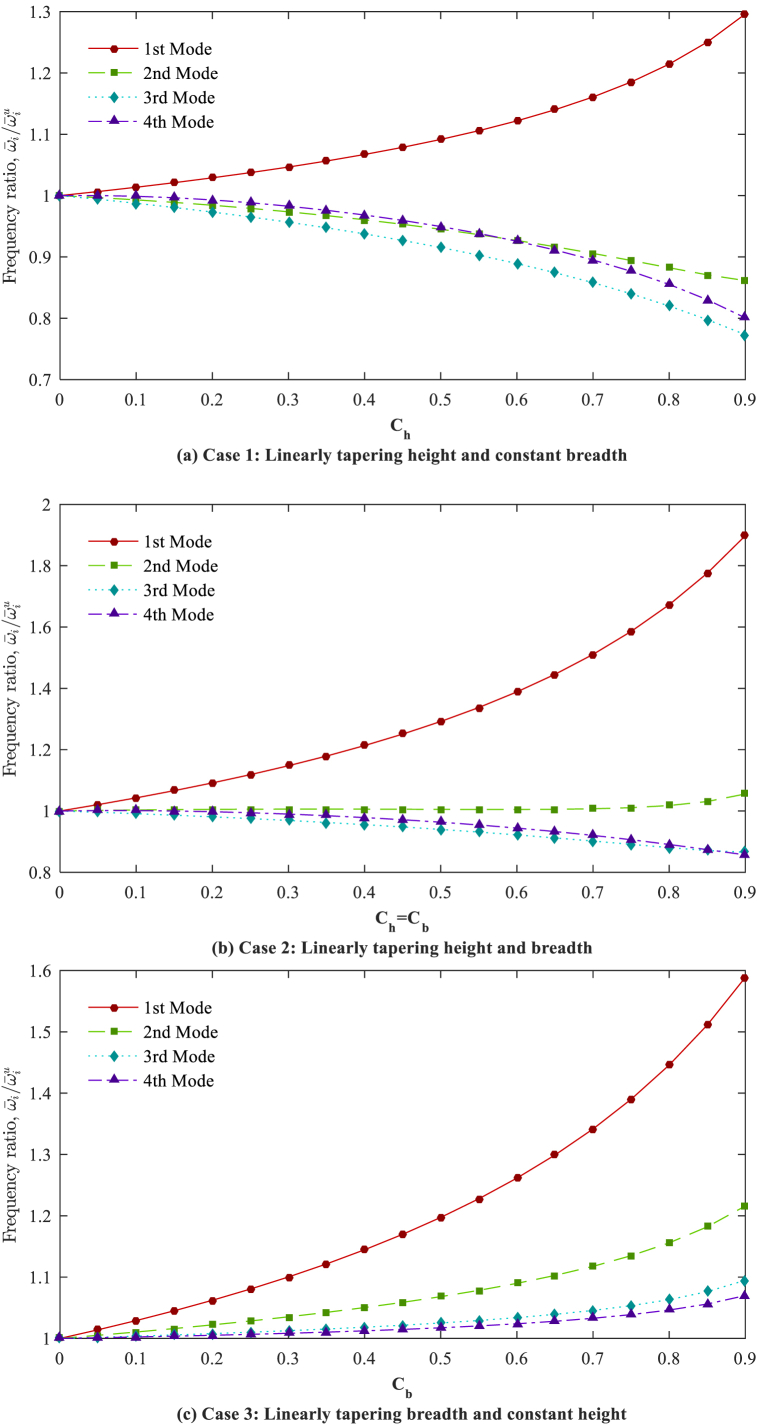
Frequency ratios ($\frac{{\bar{\omega }}_{i}}{{{\bar{\omega }}_{i}}^{u}},i=1,2,3,4$) in respect to taper ratio for different cases (*n* = 2); C-F.

**Fig. 9:**
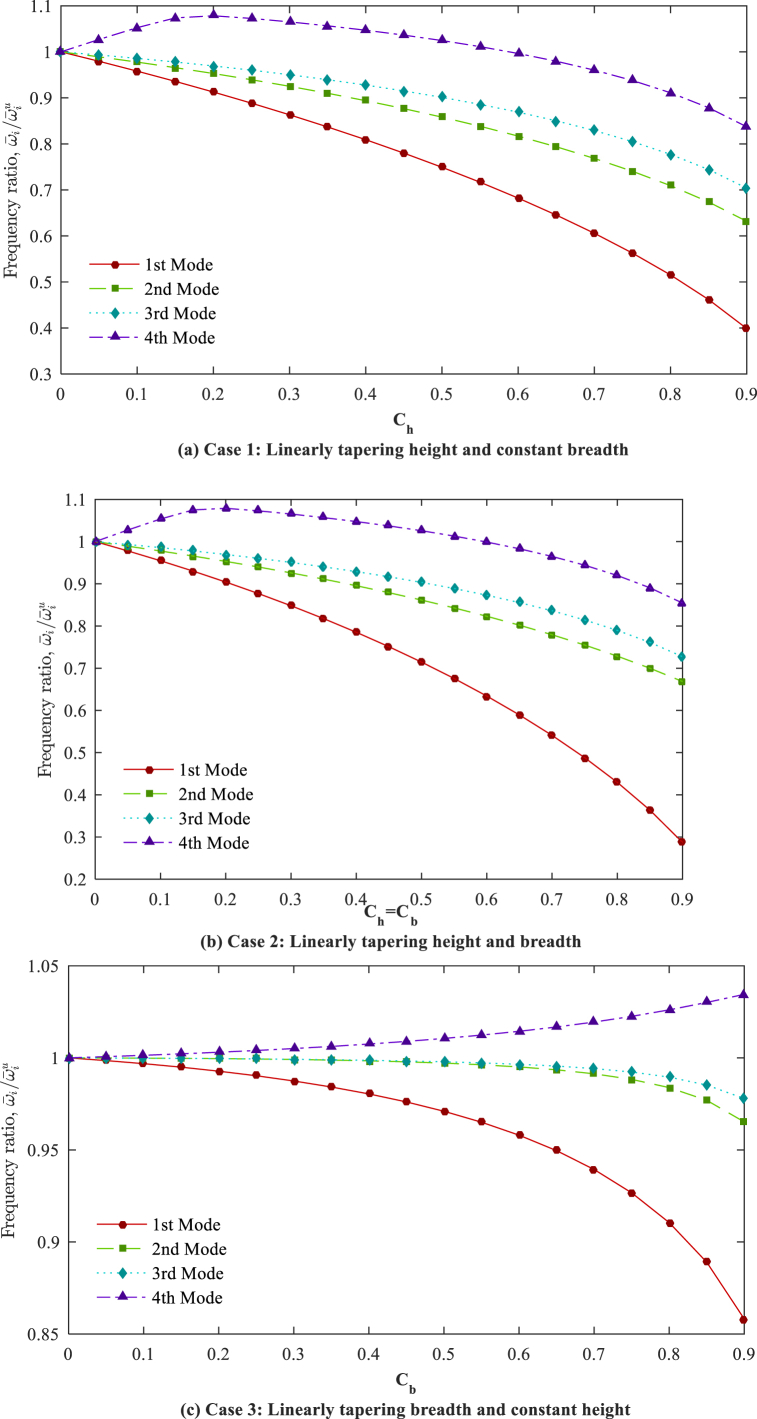
Frequency ratios ($\frac{{\bar{\omega }}_{i}}{{{\bar{\omega }}_{i}}^{u}},i=1,2,3,4$) in respect to taper ratio for different cases (*n* = 2); S-S.

**Fig. 10:**
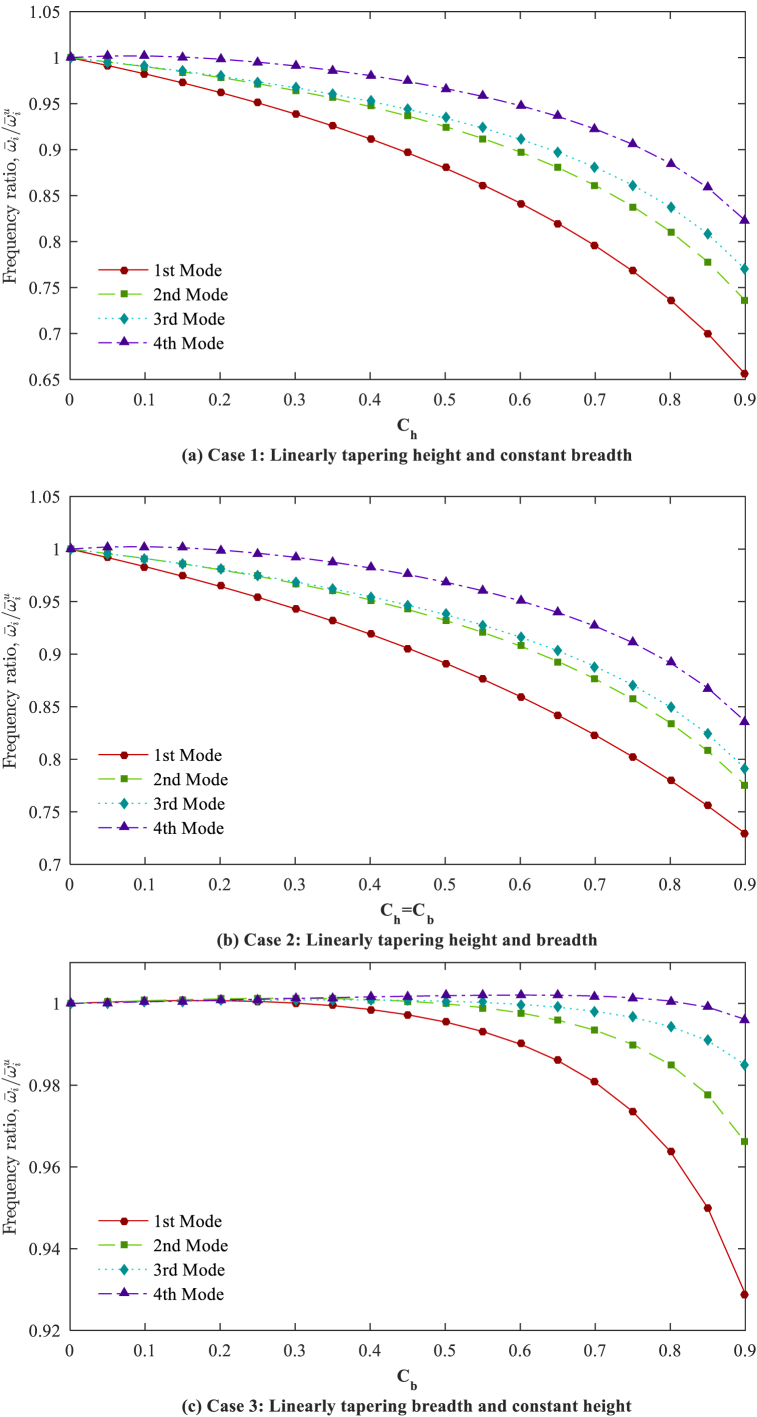
Frequency ratios ($\frac{{\bar{\omega }}_{i}}{{{\bar{\omega }}_{i}}^{u}},i=1,2,3,4$) in respect to the taper ratio for different cases (*n* = 2); C-F.

By considering a constant non-homogeneity parameter (*n* = 2), the ratio of the ${\bar{\omega }}_{i}\left(i=1,2,3,4\right)$ in the AFG tapered beams with diverse taper ratios to dimensionless frequencies in the AFG uniform beams (${{\bar{\omega }}_{i}}^{u}$) are plotted for various tapering cases and boundary conditions in Fig. 8, Fig. 9, Fig. 10. It is observed that the dimensionless natural frequencies show different variations for various tapering cases, mode numbers, and boundary conditions, indicating the considerable and unpredictable impacts of the tapering case and taper ratio.

### 3.3 Buckling

The dimensionless critical loads of AFG tapered beams are presented in Table 7 for different tapering cases, taper ratios, and boundary conditions (*n* = 2). The obtained results indicate reasonable consistency with FEM [23]. As expected, the dimensionless critical loads decrease with an increase in the taper ratio for each tapering case due to the sensitivity of the critical load to the stiffness but not to the beam's mass.

**Table 7:** Dimensionless critical loads of AFG tapered beam for different taper ratios and various boundary conditions (*n* = 2).

Taper ratio	Method	Case 1			Case 2			Case 3		
		C–F	S-S	C–C	C–F	S-S	C–C	C–F	S-S	C–C
0	Present	1.9813	5.5369	11.1989	1.9813	5.5369	11.1989	1.9813	5.5369	11.1989
0.3	Present	1.3873	3.3054	7.6755	1.1848	2.6060	5.5042	1.7344	4.4382	7.8688
	FEM	1.3879	3.3093	7.6882	1.1856	2.6104	5.6402			
0.6	Present	0.7772	1.4633	3.8946	0.4744	0.7666	1.8012	1.4290	3.1429	4.5091
	FEM	0.7784	1.4674	3.9224	0.4759	0.7707	1.8590			
0.9	Present	0.1671	0.2133	0.6658	0.0324	0.0369	0.1097	0.8758	1.1161	1.1406
	FEM	0.1701	0.2180	0.6954	0.0340	0.0390	0.1187			

*Note.* FEM: Ref [23].

**Fig. 11:**
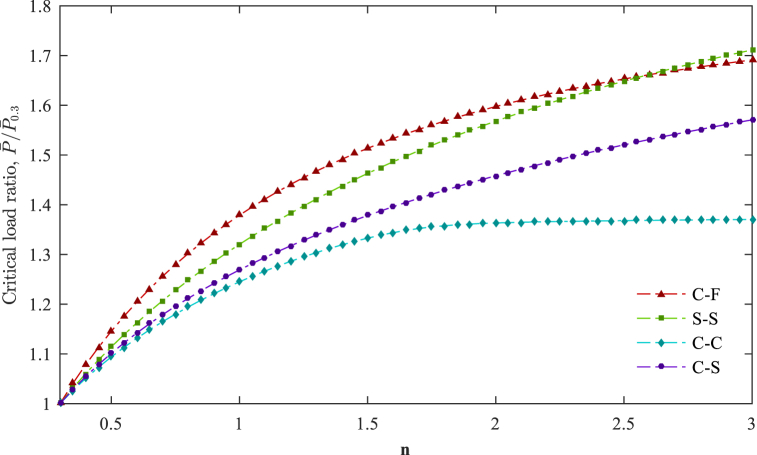
Dimensionless critical load ratio with respect to the material non-homogeneity parameter for various boundary conditions (${\bar{P}}_{0.3}=$ the dimensionless critical load with *n* = 0.3 for each boundary condition).

The buckling of AFG tapered beams is studied for various boundary conditions, and the effects of the non-homogeneity parameter and taper ratio are investigated. For more accuracy, the results are computed by 128 cubic elements. The ratio of the dimensionless critical loads of the AFG uniform beams with different material non-homogeneity parameters *n* (0.3≤n≤3) to the AFG uniform beams with *n* = 0.3 is illustrated in Fig. 11 for other boundary conditions. Due to the sensitivity of the critical load to the stiffness but not to the beam's mass, the variation of the critical load concerning *n* is predictable and increases with an increase in *n*, as shown in Fig. 11.

## 4 Conclusion

The free vibration and buckling analysis of axially functionally graded tapered Timoshenko beams are performed by applying the isogeometric method. The geometry and displacement field are modeled by B-spline basis functions, and the material properties are supposed to change axially with the power-law formula. The numerical results of axially functionally graded tapered beams with various boundary conditions, non-homogeneity parameters, mass density, Young's modulus, and taper ratios are compared with other related papers to validate the efficiency and accuracy of the isogeometric analysis method. The current findings can be used as a benchmark for future research on the axially functionally graded tapered Timoshenko beams. Moreover, our suggested approach can be applied to other future issues in axially functionally graded beams such as force vibration, non-linear vibration, and vibration on elastic foundations. Based on the numerical results, the following brief remarks are made:

• The selective reduced integration method has better results than full integration. It can efficiently mitigate the shear-locking effect, especially when the order of the basis function is low.
• The effect of the non-homogeneity parameters, taper ratio, and *E* ratio are presented For *ZrO*_*2*_*/Al* AFG beams, increasing the non-homogeneity parameter (n) makes the beam both heavier and stiffer. This can lead to unpredictable changes in natural frequencies because they are influenced by both the beam's weight and its stiffness.
• For the constant mass density ratio of ${\;\rho }_{ratio}=1$, as expected, the natural frequencies rise with an increase in *E*_*ratio*_*.* In contrast, the natural frequencies exhibited various trends for different non-homogeneity parameters and boundary conditions for $\rho_{ratio}=E_{ratio}$. It is demonstrated that the critical loads rise with an increase in the non-homogeneity parameters.
• For various taper ratios and tapering cases, it is revealed that the variation of natural frequencies for different boundary conditions cannot be predicted, whereas the critical loads decrease with an increase in the taper ratio for different tapering cases. In the first case, the beam's height varies, but its width remains fixed. In the second case, the height and width taper proportionally. In the third case, the beam's width varies, but its height remains constant.
• The critical loads, normalized for beam size, decrease when the beam becomes more tapered. This is because the beam's stiffness, rather than its mass, has a greater impact on the critical load.

## CRediT authorship contribution statement

**Farzad Abdi:** Formal analysis, Data curation, Conceptualization. **Aazam Ghasemi:** Validation, Methodology. **Alireza Ariaei:** Methodology. **S. Ali Eftekhari:** Investigation, Writing – original draft. **Mehrdad Nasr:** Formal analysis, Data curation, Conceptualization **Mohamad Khaje Khabaz:** Formal analysis, Data curation, Conceptualization. **Soheil Salahshour:** Investigation, Writing – original draft.

## Declaration of competing interest

The authors declare that they have no known competing financial interests or personal relationships that could have appeared to influence the work reported in this paper.
